# Libman-Sacks endocarditis of the mitral valve as a first presentation of systemic lupus erythematosus

**DOI:** 10.1093/jscr/rjaf989

**Published:** 2025-12-17

**Authors:** Nael Al-Sarraf, Samah AlKharji, Mohammed Hasan, Ali Alhumaidan

**Affiliations:** Department of Cardiac Surgery, Dabbous Cardiac Center, Adan Hospital, Kuwait City, Kuwait; Department of Cardiology, Dabbous Cardiac Center, Adan Hospital, Kuwait City, Kuwait; Department of Cardiology, Dabbous Cardiac Center, Adan Hospital, Kuwait City, Kuwait; Department of Cardiology, Dabbous Cardiac Center, Adan Hospital, Kuwait City, Kuwait

**Keywords:** systemic lupus erythematosus, Libamn-Sacks endocarditis, antiphospholipid syndrome, mitral regurgitation

## Abstract

Libman-Sacks endocarditis (LSE) is noninfectious verrucous vegetation lesion that can mimic infective endocarditis and the mitral valve is commonly involved. Although the exact pathogenesis remains unclear, the presence of LSE is associated with primary antiphospholipid syndrome (APS) and APS secondary to systemic lupus erythematosus (SLE). The presentation varies from mild symptoms to fulminant disease with high thromboembolic risk. Here, we present a case of LSE in female patient presented with shortness of breath and recurrent strokes but no clinical features of SLE. High index of suspicion remains a key factor in making the correct diagnosis.

## Introduction

Cardiac involvement in primary anti-phospholipid syndrome (APS) or APS secondary to systemic lupus erythematosus (SLE) ranges from 30%–80% [1]. SLE is characterized by clinical symptoms with biochemical criteria for diagnosis and in the absence of such clinical features, the diagnosis can be a challenge. Libman-Sacks endocarditis (LSE) is noninfectious verrucous vegetation lesion that can mimic infective endocarditis (IE) and the mitral valve is commonly involved with ensuing mitral regurgitation (MR). The correct diagnosis has implication on the outcomes for these patients when referred to cardiac surgery.

## Case report

**Figure 1:**
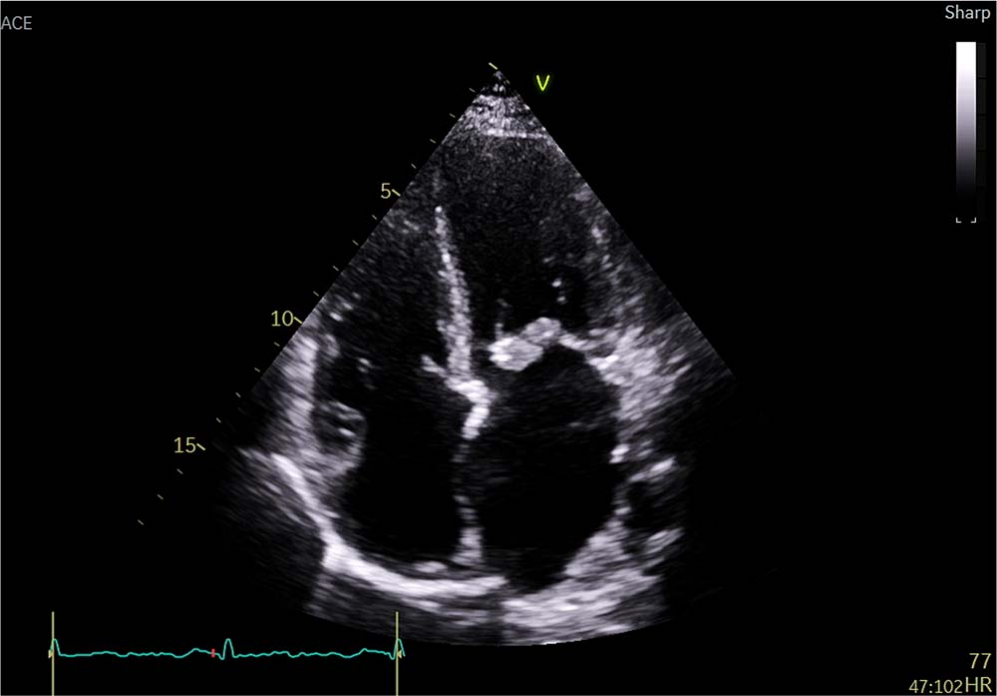
Transthoracic echo appearance of mitral valve with mass attached to posterior leaflet (apical 4 chamber view).

**Figure 2:**
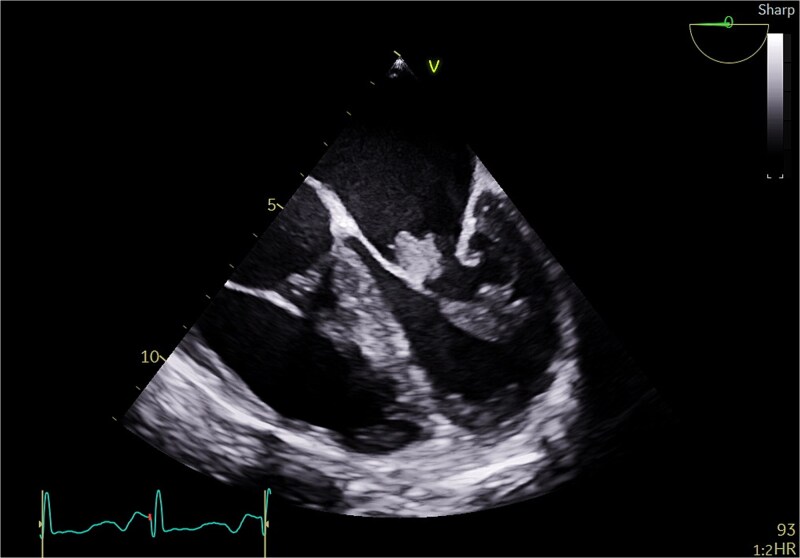
Transesophageal echo appearance of mass attached to posterior leaflet of mitral valve (mid esophageal 4 chamber view).

A 51-year-old woman with history of hypertension, hyperlipidemia, diabetes mellitus type II, previous ablation for supra ventricular tachycardia with implantation of loop recorder and sleeve gastrectomy for obesity was referred for surgical evaluation of MR. She was non-smoker with positive family history of ischemic heart disease affecting her mother. She was investigated for recurrent strokes with left sided paresthesia in 2022 and right sided paresthesia with dysarthria in 2024. She had dyspnea on minimal exertion and lower limb edema but no hemoptysis. She had one miscarriage previously with no history of deep vein thrombosis or pulmonary embolism. She had no history of myalgia, arthralgia, and no photosensitivity. Transthoracic echocardiography (TTE) showed pulmonary hypertension with moderate tricuspid regurgitation (TR) and moderate to severe MR and severe mitral stenosis (MS). The appearance was suggestive of rheumatic disease (Fig. 1). Trans esophageal echocardiography (TEE) showed severely dilated left atrium (LA) with no thrombus. Mitral valve with thickened with tethered posterior leaflet with mean gradient (MG) of 13 mmHg. Severe MS and moderate MR with large mass attached to the posterior scallops (Fig. 2) measuring 20 × 15 mm (likely thrombus). Ejection fraction was 55%. She was in sinus rhythm persistently and afebrile. Coronary angiography was normal. She was commenced on oral anticoagulation (Warfarin) for the possible thrombus, and she was reluctant for surgery. Her blood work was normal including normal complete blood count and renal, liver, and thyroid profile with therapeutic international normalized ratio (INR). Blood cultures were negative for growth. She was seen by haematology and work up revealed APS with a recommendation for lifelong warfarin. Patient subsequently accepted surgery.

**Figure 3:**
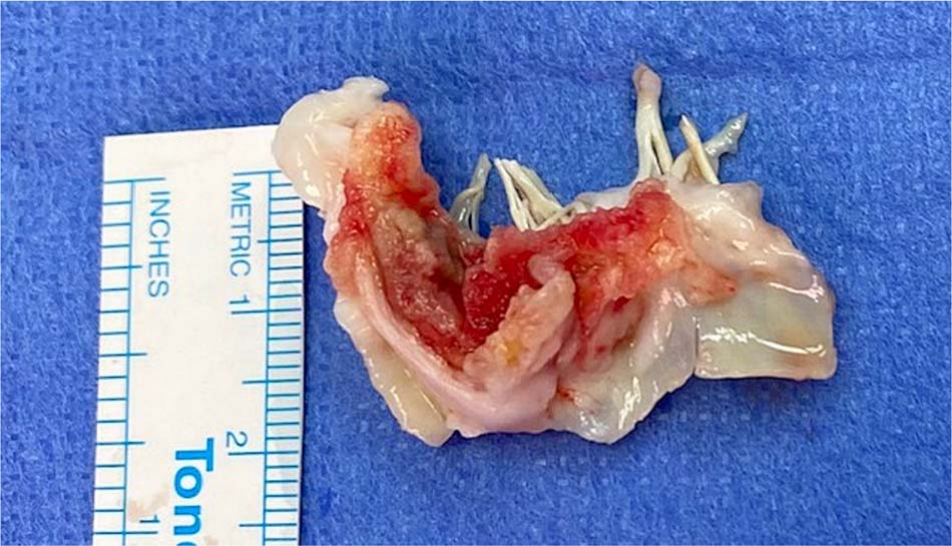
Operative view of mitral valve.

She underwent sternotomy with mechanical mitral valve replacement (MVR) and tricuspid valve (TV) repair with LA appendage clip and removal of loop recorder. Intra-operatively, there was extensive destruction of posterior leaflet and tissue damage with no dilatation of annulus (Fig. 3). Anterior leaflet was thickened. Valve was excised and sent to pathology and microbiology. Post-operative TEE showed well-functioning mitral valve with no para valvular leak and mild TR. Her post-operative course was uneventful. Cultures were sterile and histology showed chronic inflammation with fibrosis, myxoid degeneration, and calcification. Irregular tan Brown nodules were seen with areas of calcification on the mitral valve. Patient was commenced on Aspirin and warfarin. As the appearance of mitral valve was highly suggestive of LSE, the rheumatology service was consulted to rule out SLE both clinically and biochemically. Table 1 is a summary of her lab tests that confirmed the diagnosis of SLE. Pre-discharge TTE showed well-functioning mitral valve and mild TR. She was discharged home day 11 post-op with therapeutic INR. At 1 year follow up, patient was well with no strokes or bleeding.

**Table 1:** Summary of autoantibodies profile of the patient

**Test**	**Value**	**Normal Reference range**
Lupus anticoagulant	Positive	
Anti-B2GP1 IgG Anti-B2GP1 IgG (CLIA)	Positive 967.0 CU	0–20 CU
Anti-B2GP1 IgM Anti-B2GP1 IgM (CLIA)	Negative 1.3 CU	0–20 CU
ACL IgG ACL IgG (CLIA)	Positive 354.2 cu	0–20 CU
ACL IgM ACL IgM (CLIA)	Negative 7.9 CU	0–20 CU
Anti-phospholipid antibody screen	Positive	
ANA screen and pattern	Nuclear and cytoplasmic positive Speckled (AC-4,AC-5) and cytoplasmic speckled (AC-19, AC-20)	
ANA titer	1:160	
Anti-ds-DNA antibody Anti-ds-DNA antibody (CLIA)	Positive 48.7 IU/ml	0–35 IU/ml
Anti-extractable antigen antibody (Anti-ENA antibody) panel	Negative	
Anti-Myeloperoxidase Ab (MPO)	Negative (0.8 IU/ml)	0–5 IU/ml
Anti-proteinase-3 antibody	Negative (1.3 IU/ml)	0–10 IU/ml

Anti-B2GP1: anti-beta-2 glycoprotein 1 antibody; ACL: anti-cardiolipin antibody

ANA: anti-nuclear antibody; CU: Clinical unit; IU: international unit; ML: milliliter

## Discussion

APS is an autoimmune disease that can be primary or secondary to SLE (40% of cases). The main features are arterial or venous thrombosis, recurrent abortions, and specific antibodies detection. These autoantibodies are directed against plasma protein such as Beta-2 glycoprotein 1 (B2GP1) or prothrombin and depend on negatively charged phospholipids. Other symptoms such as thrombocytopenia, livedo reticularis, seizures, valve disease, or organ failure occur in both forms of APS [2]. LSE is noninfectious verrucous vegetation lesion that mimic IE. Mitral and aortic valves are the commonest involved. It is typically mild and asymptomatic but can lead to fulminant presentation such as superimposed bacterial endocarditis, thromboembolic events, and heart failure [3, 4]. The definitive diagnosis can be achieved by histology which shows fibrin deposits, fibroblastic organization, neovascularization, immune complexes, and infiltration of mononuclear cells. In the healed form, there is fibrous plaque with focal calcification, marked scarring, and valve deformity/destruction [4–6]. The pathogenesis is likely immunological as suggested by the presence of immunoglobulins and complement on the affected valves. About 30%–40% of patients with primary APS and 50%–60% of patients with SLE show an affection for one or more valves. Valve operation is performed at age between 25 and 55 years old with female: male ratio of 3:1. Thrombotic/thromboembolic events (rather than foetal loss) are the typical feature in APS patients with valve disease. LSE appears echocardiographically as masses of varying size and shape, sessile, and firmly attached to valve surface with irregular borders. They can occur at the commissures, free margins, and annulus. Left sided valves are commonly affected with MR most seen [3, 5]. Valve choice is controversial. The young age and the need for long-term anticoagulation for APS makes mechanical valve a first option. However, the advantage of bioprosthesis is the freedom form anticoagulation but numerous reports showed early bioprosthetic valve degeneration [2]. Surgical mortality rates can reach up to 33% [7–9] with significant bleeding and thromboembolic risks (Table 2). Poor prognostic factors reported were presence of MR, LSE, low Complement-3 level and higher steroid dose but not the type of vale used (mechanical vs biological) [1].

**Table 2:** Surgical outcomes of valve surgery in antiphospholipid syndrome and systemic lupus erythematosus

**Author/year**	**Number of patients**	**Surgical mortality**	**Complication rate**	**Comment**
Eviatar/2023	26	15.4%	54%	Cardiogenic, valve thrombosis, stroke, major bleeding, valve infection Redo surgery (11.5%)
Erdozain/Ref 16 above artcile	32	12.5%	50%	Infection, thrombosis, hemorrhage and cardiogenic (44%)
Arif (ref 31) Eviatar article	15	Zero with 20% mortality at 4 years	60%	Cardiovascular and thromboembolic events
Colli (Ref 22 Eviatar article)	9	33%	67%	Thromboembolic, haemorrhagic events

Our case was unusual in many ways. Firstly, the presentation was multiple strokes with absence of SLE symptoms. Secondly, there was no physical signs of SLE which was only proved by biochemical testing and valve appearance intraoperatively. Finally, the combination of LA appendage occlusion with mechanical MVR was a successful strategy with good prognosis. We have closed LA appendage to minimize her future risk of stroke from embolic source.

## Conclusion

LSE with associated MR can be a rare presentation for SLE in a young female patient with absence of associated clinical signs of SLE. The combination of mechanical mitral valve replacement with occlusion of LA appendage can be a valuable strategy to prevent the associated embolic stroke risk.
